# *Wherever I May Roam*: A Time-Resolved Wavelength-Dependent Study of the Roaming Dynamics in Acetaldehyde

**DOI:** 10.1021/acsearthspacechem.6c00134

**Published:** 2026-06-23

**Authors:** Derri J. Hughes, Michael A. Parkes, Richard T. Chapman, M. Nrisimhamurty, Emma Springate, James O. F. Thompson, Tiffany Walmsley, Yu Zhang, Russell S. Minns

**Affiliations:** † 7423 School of Chemistry and Chemical Engineering, University of Southampton, University Road, Highfield, Southampton SO17 1BJ, U.K.; ‡ Department of Chemistry, 4919 University College London, 20 Gordon Street, London WC1H 0AJ, U.K.; § Central Laser Facility, 97008 STFC Rutherford Appleton Laboratory, Didcot, Oxfordshire OX11 0QX, U.K.

## Abstract

Acetaldehyde represents a key carbonyl compound in star-forming regions of the Sagittarius B2 molecular cloud, where photolysis generates reactive intermediates central to astrochemical complex molecule formation. Yet, its dominant photodissociation mechanisms remain poorly understood. Here, we use extreme ultraviolet (21.5 eV) time-resolved photoelectron spectroscopy, supported by high level *ab initio* calculations, to investigate the wavelength-dependent photodissociation dynamics of acetaldehyde following excitation at 295 and 308 nm. At 295 nm, population of the S_1_ $\left(n_{O}\to \pi_{C=O}^{*}\right)$ state decays on a 60 fs time scale via an energetically accessible S_1_/S_0_ conical intersection seam, leading to both direct dissociation *via* conventional transition state dynamics and the formation of longer-lived (4.0 ps) CH_3_ roaming intermediates on S_0_. These roaming intermediates subsequently yield radical and molecular products over a hundreds-of-picoseconds time scale. In contrast, excitation at 308 nm produces low intensity structure between 7.0–8.5 eV at intermediate and late delay times, which, together with EOM-IP-CCSD calculations of representative geometries, suggest that additional pathways, such as H-atom roaming, may be active, leading to acetyl radical formation on a tens-of-picoseconds time scale. These findings demonstrate that access to competing S_1_ pathways in acetaldehyde may be strongly wavelength- and mode-dependent, controlling the balance between conventional dissociation and distinct roaming mechanisms.

## Graphical abstract

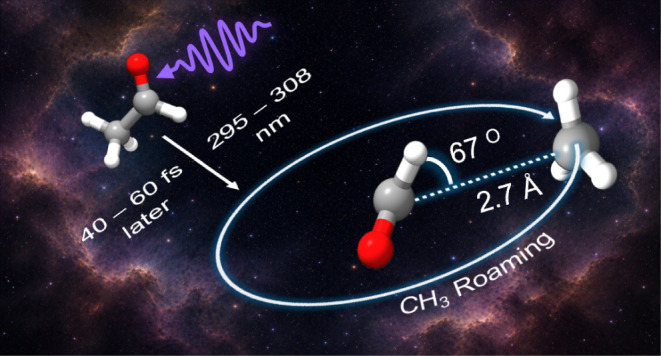

## 1 Introduction

The Sagittarius B2 (Sgr B2) molecular cloud, located approximately 390 light years from the Galactic Center, is one of the most chemically diverse regions of the interstellar medium (ISM). Its rich molecular inventory,^1–3^ active star formation,^4,5^ and extensive spectroscopic coverage^3,6,7^ make it a central target for modern astrochemical studies. Among the “zoo” of complex organic molecules detected in Sgr B2 is acetaldehyde (CH_3_CHO), first observed in 1973.^8^ Acetaldehyde’s astrochemical importance stems from its role as a tracer of ultraviolet (UV) and cosmic-ray-driven chemistry in molecular clouds and hot cores,^9^ and as a precursor of prebiotic molecules such as amino acids and sugars through both gas-phase and solid-state reaction pathways.^10,11^ Although the dense interior of Sgr B2 offers some shielding from external radiation, internal UV photons produced by young protostars can still drive significant photochemistry.^12–16^ For acetaldehyde, the absorption of a UV photon within the 230–340 nm range initiates an $n_{O}\to \pi_{C=O}^{*}$ transition, leading to dissociation into HCO + CH_3_ and CO + CH_4_ product pairs.^17–28^ In modern astrochemical models, this reaction is described *via* wavelength-dependent photodestruction rates, which necessarily simplify the underlying excited state dynamics.^29,30^ Yet these dynamics determine product branching ratios and internal energy partitioning—quantities that directly affect radical reactivity and the predicted chemical evolution of the ISM. Herein, we present the results of a wavelength-dependent study of the photodissociation of acetaldehyde at 295 and 308 nm, focusing on the underlying excited state dynamics and how they drive photodissociation.

Despite the seemingly simple picture of dissociation described above, acetaldehyde’s excited state dynamics leading to dissociation are far from it. Over the past two decades, experimental and theoretical work has revealed an alternative dissociation pathway that competes with conventional transition-state-mediated dissociation: the roaming mechanism. In the traditional picture of unimolecular reactions, molecules traverse a well-defined transition state (TS) en route from reactants to products. Roaming proceeds when partially dissociated fragments form a long-range, quasi-bound complex that explores flat regions of the potential energy surface (PES), sampling multiple different geometric configurations before producing stable products.^18,19,22,25,26,31^ Energy-resolved spectroscopic studies of the fragments have captured characteristic signatures of roaming. These include bimodal energy distributions and unusual internal energy partitioning.^19–23,27,28,31^

A central challenge in understanding roaming lies in characterizing its transient intermediates. These structures, by their very nature, do not correspond to well-defined points on the PES, making them elusive and potentially fleeting. Their experimental identification has, so far, required an ultrafast universal probe capable of ionizing all transient species—regardless of their ionization energies. Time-resolved Coulomb explosion imaging provided the first direct experimental evidence for these intermediates in deuterated formaldehyde. Endo *et al.* identified a distinct set of reaction intermediates with kinetic energy releases (5–11 eV) and characteristic angular momentum correlations that were inconsistent with conventional molecular or radical dissociation alone.^25^ Comparison with simulated roaming trajectories supported the assignment of these reaction intermediates to a loosely bound deuteron exploring the ground state potential energy surface before dissociating or abstracting the second deuteron—all on sub-picosecond time scales.^25^ More recently, Abma *et al.* employed time-resolved photoelectron spectroscopy (TRPES) with a universal extreme ultraviolet (XUV) probe to observe roaming intermediates in acetaldehyde, supported by complete active space self-consistent field (CASSCF) and equation-of-motion ionization potential coupled-cluster (EOM-IP-CCSD) calculations.^26^ Following excitation at 262 nm, they reported ultrafast decay of the first excited singlet (S_1_) state on a time scale of ∼50 fs *via* an S_1_/S_0_ conical intersection (CI) accessed along the C–C bond extension and CCH bending coordinates. Photoelectron features assigned to roaming intermediates appeared in the 5.3–7.6 eV binding energy range and persisted for up to 190 ps, while HCO signals, attributed to both ultrafast TS-mediated dissociation on S_1_ and from roaming processes on the ground state (S_0_), exhibited no measurable decay over 600 ps. The roaming assignments were supported through comparison of the observed binding energies with EOM-IP-CCSD calculations of roaming geometries, in which a CH_3_ roams about an HCO core, sampled from their S_0_ PES.

Despite this recent progress, direct spectroscopic investigations of acetaldehyde photodissociation at longer UV wavelengths remain limited and somewhat contradictory. Figure 1a provides reaction schemes for the various pathways leading to the formation of the molecular products and their reported branching ratios at 308 nm.^22,27,28^ Early laser-induced fluorescence measurements by Houston and Kable suggest that at 308 nm, dissociation via the first excited triplet state (T_1_) TS (i) competes with roaming processes.^19^ However, later work at the same wavelength suggests that CH_3_ roaming (ii) is in fact the dominant process by which photodissociation occurs.^22,28^ Interestingly, Heazlewood and coworkers reported evidence from quasi-classical trajectory simulations for a second roaming mechanism that could not be quantified in their infrared measurements of the molecular photodissociation product, CH_4_.^22^ Lee *et al.* later succeeded in quantifying this additional roaming pathway through the observation of a new cold component in the CO (ν = 0) product distributions.^28^ While both studies established a dominant CH_3_ roaming mechanism, Lee *et al.* identified a minor pathway (iii)—accounting for 13% of the total CO product yield—in which an H atom “wanders” about a CH_3_CO core (H-atom roaming), analogous to that previously observed in formaldehyde.^31^

**Figure 1:**
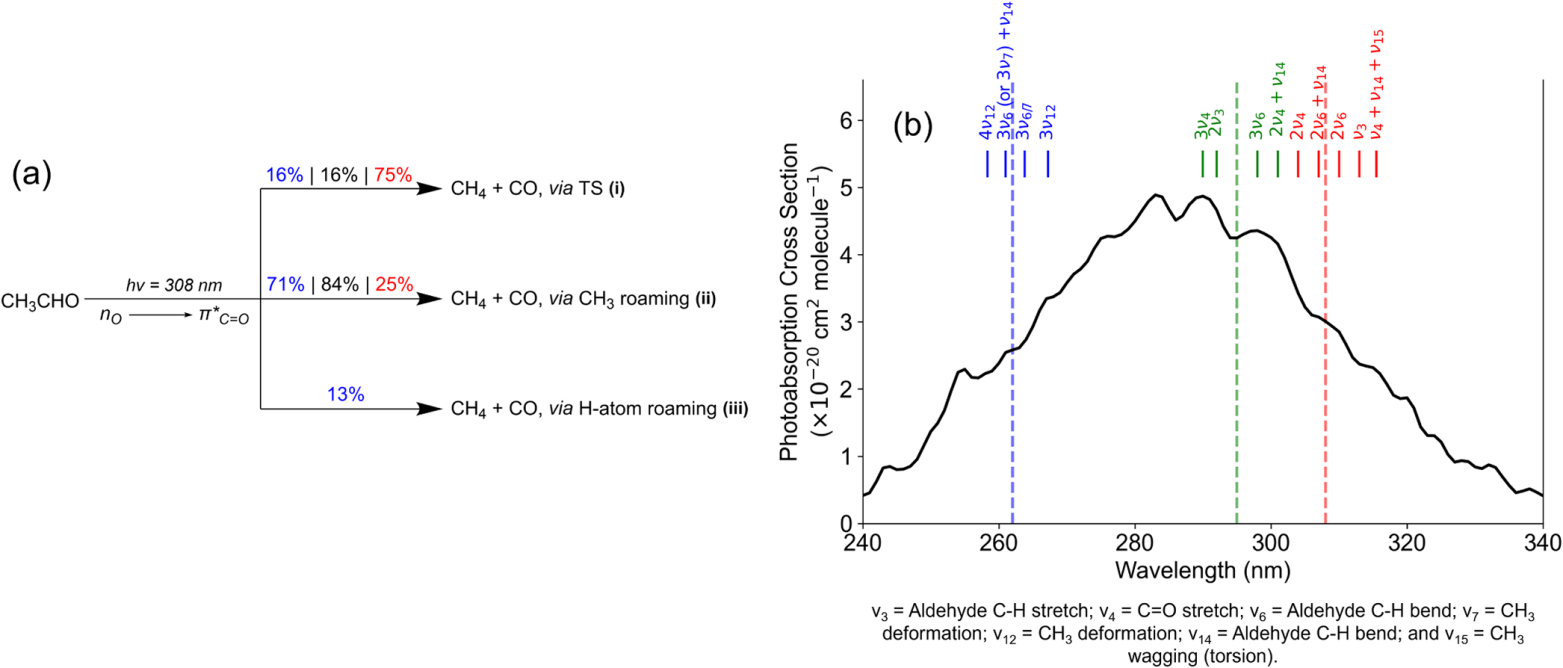
(a) Schematic reaction equations for the formation of the molecular products, CH_4_ and CO, in the photodissociation of acetaldehyde at 308 nm. Blue branching ratios are from ref 28, black from ref 22, and red from ref 27 (b) Absorption spectrum of acetaldehyde over the $n_{O}\to \pi_{C=O}^{*}$ band, indicating the wavelengths studied by XUV-TRPES by Abma et al.^26^ (blue dashed line, 262 nm) and in this work (green and red dashed lines, 295 and 308 nm). The vibronic states within 5 nm around each excitation wavelength are also given, with a key of the vibrational modes below. Reproduced from refs 17 and 32 with permission from Elsevier.

In contrast, CO ion imaging measurements by Rubio-Lago *et al.* reported a substantially higher branching ratio for the TS mechanism (75%, (i)), with a corresponding roaming contribution of 25% (ii).^27^ Supported by CASSCF calculations with second-order perturbation theory corrections at selected points on the PES, these authors interpreted their results as evidence for dynamics occurring below the S_1_/S_0_ CI, leading to a dominant TS mechanism on the T_1_ state, in agreement with the earlier conclusions of Houston and Kable.^19^ Although a roaming contribution was identified in their measurements, it was attributed solely to the CH_3_ roaming mechanism (ii) with no discussion of a possible H-atom roaming pathway (iii). It is also important to note that these measurements were sensitive only to molecular products; as a result, the branching ratios between radical and molecular fragmentation channels remain unknown, particularly for the acetyl radical (CH_3_CO), which is believed to play a key role in the H-atom roaming channel. While all of these experiments suggest that roaming plays some role in the photodissociation at 308 nm, they disagree on its relative importance and can only indirectly assign the underlying reaction mechanisms on the basis of product state distributions.

While recent measurements at shorter UV wavelengths (262 nm) by Abma *et al.* directly observe CH_3_ roaming, these results suggest that multiple roaming pathways may be accessible and may compete with TS mechanisms at longer UV wavelengths.^26^ This has important astrochemical implications as distinct product channels can initiate different gas-phase reactions and influence chemical evolution. Consequently, two overarching questions remain: *what are the dominant dissociation mechanisms at longer UV wavelengths, and how are they accessed following excitation to S*_*1*_? To address these questions, we employ XUV-TRPES to investigate the photodissociation of acetaldehyde initiated at 295 and 308 nm. By direct comparison with the 262 nm study of Abma *et al.*, we establish how roaming dynamics evolve across the center of the $n_{O}\to \pi_{C=O}^{*}$ absorption band, as shown in Figure 1b.

## 2 Methodologies

### 2.1 Experimental Section

Time-resolved photoelectron spectroscopy measurements were performed using the Atomic, Molecular, and Optical (AMO) endstation at Artemis at the Central Laser Facility. An in-depth experimental setup is given elsewhere,^33^ with only a brief overview given below.

Pump and probe pulses were generated from the output of an amplified Ti:sapphire system (Red Dragon, KM Labs) operating at a central wavelength of 800 nm and a 1 kHz repetition rate. The output was split, providing separately compressed pulses used to generate the pump and probe. The 295 and 308 nm pump pulses were generated by an optical parametric amplifier (TOPAS-HE-PRIME, Light Conversion) via the fourth harmonic of the signal, yielding pulse energies of 15 μJ at both wavelengths. Pulse durations, obtained from our fitted Gaussian instrument response functions (IRF), were 70 ± 10 and 60 ± 7 fs at 295 and 308 nm, respectively. The resultant pump pulses were focused to a beam spot of approximately 100 μm (fwhm) in the pump–probe interaction region. XUV probe pulses were generated via high harmonic generation (HHG) in an Ar gas jet. The HHG process was driven by the second harmonic (400 nm) of the fundamental, with a quasi-time-preserving monochromator subsequently used to select the seventh harmonic of the driver (nominally 21.5 eV, 57.4 nm). The isolated harmonic has a nominal flux of 10^10^ photons per second on target with a pulse duration of approximately 35 fs.^34^ Pump and probe pulses were independently propagated, focused, and overlapped by a small angle (∼3°) at the interaction point of the electron time-of-flight spectrometer and the molecular beam.

The molecular beam was generated by expanding approximately 1 bar of 2% acetaldehyde in helium (BOC specialty gases) through the 200 μm nozzle of a piezoelectric valve (Amsterdam Piezovalve, ACPV2) operating at a 1 kHz repetition rate. The resultant molecular beam was skimmed (0.8 mm, Beam Dynamics) once before entering the detection chamber. The liberated photoelectrons were detected using an electron time-of-flight spectrometer (Kaesdorf ETF11), in which a conical electrostatic lens defines the angular and kinetic energy acceptance of the instrument (∼5% collection efficiency). Experimental runs at both 295 and 308 nm were performed with an applied voltage of 95 V to optimize the transmission of photoelectrons within the expected kinetic energy range of 12–16 eV, associated with the initially excited state and low binding energy intermediates and products.

Time-resolved photoelectron spectra were collected as a function of pump–probe delay (−1 to +300 ps at 295 nm; −0.6 to +300 ps at 308 nm), where negative delays correspond to the XUV pulse arriving before the UV pulse and positive delays to the reverse. Time zero (*t*_0_) is defined as the point of maximum pump–probe temporal overlap. The spectra were calibrated against photoelectron signals reported by Tam et al.,^35^ and the calibrated spectra are provided in the Supporting Information (Figure S1).

### 2.2 Supporting Calculations

To support our experimental observations, we performed state-averaged CASSCF (SA-CASSCF) calculations using Molpro^36,37^ to characterize the excited state landscape of acetaldehyde, and EOM-CCSD and EOM-IP-CCSD calculations using ORCA 6.0.0 ^38–40^ to estimate excitation and ionization energies for key ground and excited state geometries. The 6-311G** basis set was used throughout.^41^

The active space comprised 14 electrons and 13 orbitals, selected based on second-order Møller–Plesset perturbation theory (MP2) natural occupations. In concurrence with previous work,^24,26^ the active space included the *n*_O_, $\pi /\pi_{C=O}^{*}$ , $\sigma /\sigma_{C-C}^{*}$ , and four $\sigma /\sigma_{C-H}^{*}$ orbitals. The ground state geometry was optimized at the CASSCF­(14,13) level and confirmed as a true minimum by subsequent frequency analysis (no imaginary modes; see Table S2 in Supporting Information). The computed vertical excitation energy for the $n_{O}\to \pi_{C=O}^{*}$ transition was evaluated to be 4.52 eV, at the 2SA-CASSCF­(14,13) level, which is in agreement with experimental values^17^ and EOM-CCSD values (4.36 eV). Starting from the ground state optimized structure, the equilibrium structure of the S_1_ state was subsequently optimized at the 2SA-CASSCF­(14,13) level. Two-dimensional (2D) rigid scans of the PES were carried out along the C–C bond and CCH bending coordinates at the 2SA-CASSCF­(14,13)/6-311G** level. One-dimensional (1D) PES cuts along the C–C and C–H bond coordinates on S_0_, S_1_, and T_1_ were evaluated at the 3SA-CASSCF­(14,13) level, from which spin–orbit coupling (SOC) matrix elements between all three states were obtained.

To aid in the interpretation of the experimental data, EOM-IP-CCSD calculations were performed on the S_0_ and S_1_ equilibrium structures, critical roaming geometries previously reported by Bowman *et al.*,^42^ and an MP2-optimized acetyl radical geometry.

## 3 Results and Discussion

### 3.1 Experimental Results

#### 3.1.1 λ*_pump_* = 295 nm

In Figure 2a, we present the time-resolved photoelectron spectrum of acetaldehyde recorded with a pump wavelength of 295 nm. The onset of the ground state ionization signal is observed at approximately 8.5 eV, above which we cannot isolate a clear pump–probe signal due to the poor pump–probe signal-to-noise ratio. Initially, we observe a short-lived signal in the binding energy range of 5.7–7.0 eV which decays on an ultrafast time scale into a broad and long-lived signal whose maximum intensities are contained within the binding energy range of 7.5–8.5 eV. Upon closer examination of how the spectrum evolves over discrete pump–probe delay time ranges, as shown in the time-averaged photoelectron spectra in Figure 2b, the temporal evolution of specific spectral features can be disentangled. In black, the pre-time-zero spectrum is plotted over the 5.5–9.0 eV binding energy range which displays our XUV-only spectrum (pre-*t*_0_) and our spectral noise baseline within this region. At delays around time zero (red trace, −0.15 to +0.15 ps), we observe a broad continuum of binding energies covering the 5.7–8.5 eV range. At intermediate delays (green trace, +0.6 to +6 ps), the low binding energy wing of the initial signal decays, narrowing the overall signal trace, and the spectrum becomes dominated by signals over the 6.8–8.5 eV binding energy range. These ionization signals appear to be reduced in intensity toward late times (blue trace, +100 to +300 ps), largely dominated by ionization signals around 8.0 eV.

**Figure 2:**
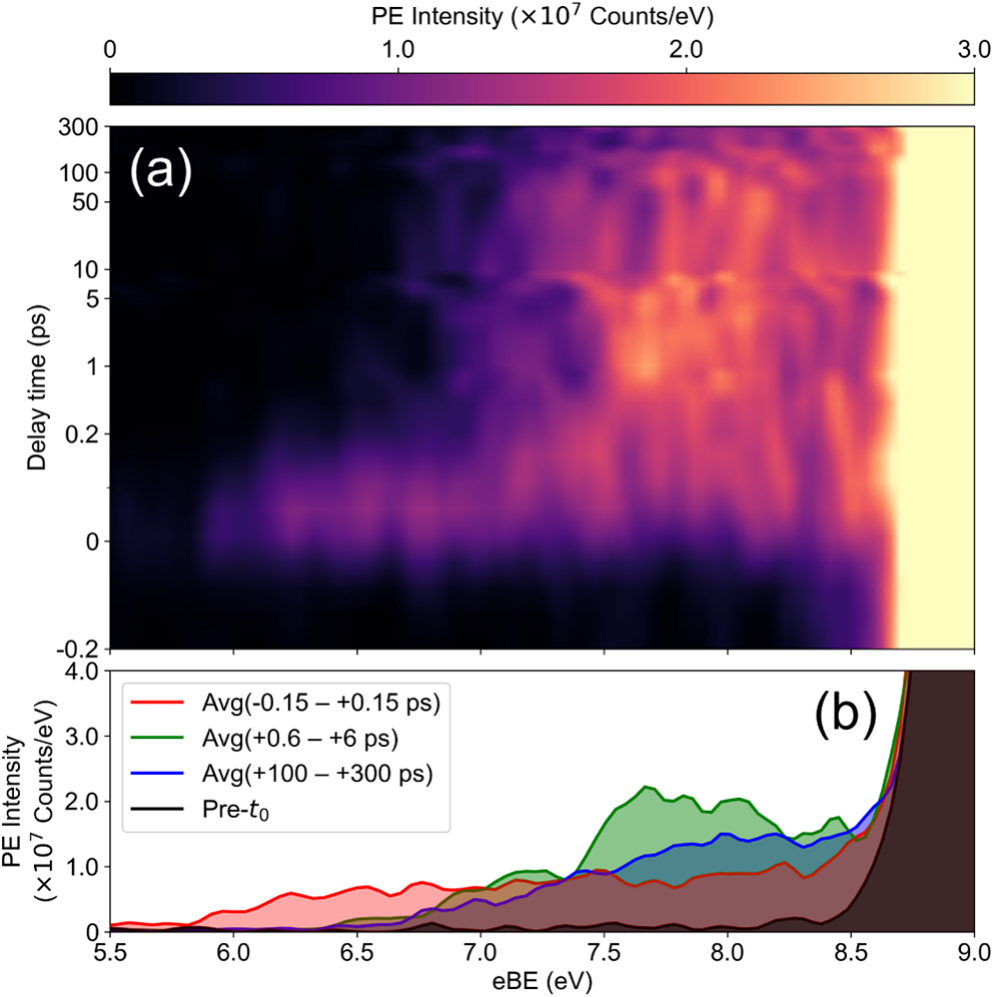
Time-resolved (a) and time-averaged (b) photoelectron spectra of acetaldehyde recorded with a pump wavelength of 295 nm. Note that the delay time axis in (a) is plotted on a mixed linear (up to +0.2 ps)-logarithmic scale. The traces in (b) are averaged (Avg) over specific pump–probe delay time ranges and plotted over the 5.5–9.0 eV binding energy region. All data have a Gaussian filter applied, with a sigma of 1, to highlight the general trend in the flow of signal intensity (population). The acronyms eBE and PE mean electron binding energy and photoelectron, respectively.

To extract the time scales upon which the dynamics occur, a 2D global fit over the 5.5–8.5 eV binding energy range was performed. This energy range was selected to obtain a good low binding energy baseline and to avoid the onset of the ground state acetaldehyde signal. The time-resolved photoelectron spectrum was modeled using a Gaussian IRF,

1 $$g\left(t\right)=A_{\mathrm{IRF}}\exp\left[-{\left(\frac{t-t_{0}}{2\sigma_{\mathrm{IRF}}}\right)}^{2}\right]$$

centered at *t*_0_ with width σ_IRF_. This IRF was convolved with a sum of exponential decay terms to give the full model:

$$S\left(eBE,t\right)=\underset{i}{\sum }A_{i}\left(eBE\right)e^{-\lambda_{i}(t-t_{0})}⊗g\left(t\right)$$ 2

where λ_*i*_ are the decay constants (and the *i* ^th^ time constant (τ_*i*_) is equal to 1/λ_*i*_) and *A*_*i*_(*eBE*) are the binding energy-dependent amplitudes. Further details of the fitting routine and residual analysis are provided in Supporting Information (Section S2).

The resultant decay-associated spectra (DAS) represent spectral components associated with distinct time constants relative to the fitted time zero. Within a given DAS, regions of positive amplitude correspond to a decaying population, while negative amplitudes indicate a rising population on the same time scale, enabling the flow of population between electronic or configurational states to be mapped.^43^

Figure 3 shows the DAS (a) and corresponding fits to the integrated intensities (b–c) over −1 to +2 ps, capturing the decay of the initial excited state and the rise of the longer-lived signal, from which three time components are extracted. The reported bounds on each fitted parameter correspond to the symmetric 95% confidence level obtained from residual bootstrap analysis (more details in Supporting Information, Section S3 ^45^). The first spectral component has a time constant of 60 ± 25 fs ( $\tau_{S_{1}}$ , black in (a)) and consists of a broad positive contribution, centered at 6.3 eV which covers the binding energy range of 5.5–7.5 eV, and a negative contribution over 7.5–8.1 eV. Due to the energy appearance of the positive component relative to the ground state acetaldehyde signal (10.22 eV, see Figure S1) and the expected binding energy shift associated with excitation to S_1_ by a 4.2 eV photon, we assign the positive contribution to ionization from the S_1_ state and the associated time constant to its decay. The negative contribution of this signal therefore indicates population transfer from S_1_ into an intermediate or product state within the 7.4–8.1 eV region.

**Figure 3:**
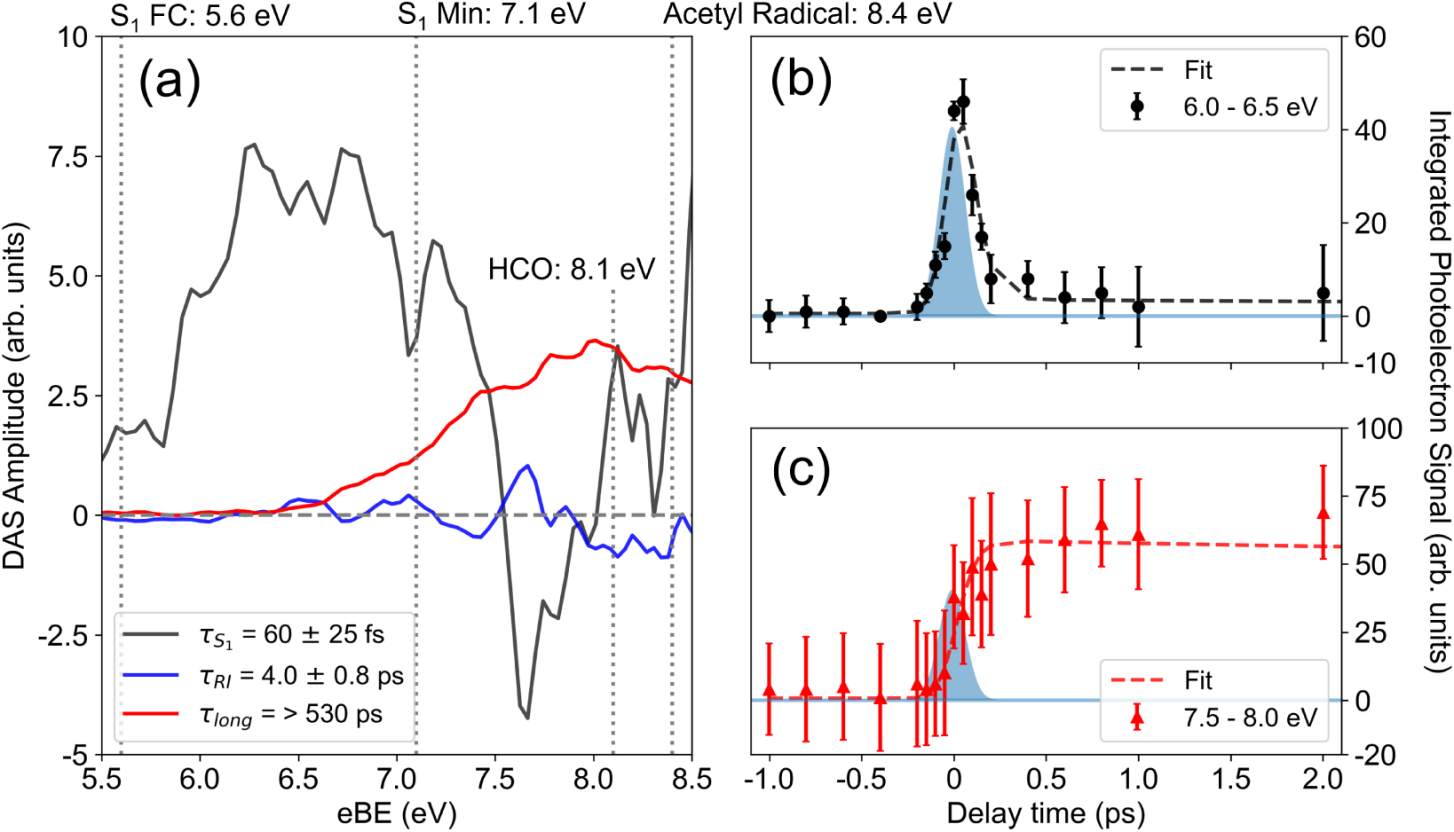
Decay-associated spectra (DAS) and extracted time constants (a), and the corresponding integrated intensity profiles (b–c), for the time-resolved photoelectron spectrum recorded at 295 nm (Figure 2a). Vertical gray dashed lines in (a) indicate the binding energies of the S_1_ Franck–Condon (S_1_ FC) and equilibrium (S_1_ Min) geometries (SA-CASSCF­(14,13), ionization energies calculated at the EOM-IP-CCSD/6-311G** level), as well as expected products: the HCO radical (literature value from ref 44) and the acetyl radical (EOM-IP-CCSD/6-311G**). In panels (b) and (c), integrated intensity profiles are shown over the delay range −1 to 2 ps, extracted from energy regions of the DAS with minimal overlap between components. Shaded regions represent 95% confidence intervals at each delay time point. All uncertainties were obtained via bootstrap analysis. Further details are provided in the Supporting Information (Section S3).

The second component (τ_RI_, blue in (a), where RI denotes the reaction intermediate) appears within a narrow binding energy range (7.5–7.8 eV) of the negative contribution of $\tau_{S_{1}}$ . Although this feature cannot be straightforwardly assigned on the basis of its energy range or lifetime alone, the clear sign of inversion between $\tau_{S_{1}}$ and τ_RI_ DAS components suggests that decay of the S_1_ state feeds the population of this region. This behavior is consistent with the formation of some reaction intermediate, accessed via the decay of the S_1_ state, which subsequently decays over 4.0 ± 0.8 ps. While the spectral range overlaps with that of CH_3_ roaming intermediates reported by Abma *et al.*,^26^ the population of a low-lying triplet state via intersystem crossing (ISC) cannot be excluded on spectral or temporal grounds alone. Therefore, a definitive assignment of this feature is deferred. Interestingly, a small negative contribution is also observed in τ_RI_ over the 8.0–8.4 eV region, suggesting that the decay of this reaction intermediate drives the population of another intermediate or product.

The final time constant (τ_long_ > 530 ps, red in (a)) exceeds the maximum pump–probe delay scanned (+300 ps) and should therefore be considered as infinite, with no measurable decay within the experimental time window. This component is assigned to the formation of a reaction product that accumulates over the observed time scale, with any subsequent decay occurring beyond the delay range probed here. On this time scale, the formation of both radical (HCO and CH_3_) and molecular (CO and CH_4_) photolysis products is expected.^26^ Of these, the only photolysis product observable in this spectral region is the HCO radical in its ground state, which has an expected ionization energy of 8.1 eV.^44^ The maximum of this DAS component corresponds closely to the known HCO ionization energy, and we therefore assign it accordingly. All other photolysis products, including CH_3_, CO, and CH_4_, are expected to appear in the 9.8–14 eV binding energy range^46–48^ where they are obscured by strong ground state acetaldehyde signals (see Figure S1), making confident assignment challenging. A major proportion of the positive amplitude of τ_long_ overlaps with the negative components of $\tau_{S_{1}}$ and τ_RI_, further suggesting that HCO formation occurs on an ultrafast time scale directly from the decay of S_1_ and on a longer time scale from the decay of the reaction intermediate.

Regions of the DAS that are relatively free from mutual overlap define discrete integration windows in which the quality of the fits can be assessed. These regions are 6.0–6.5 eV $\left(\tau_{S_{1}}\right)$ and 7.5–8.0 eV (τ_RI_ and τ_long_), and are shown in Figure 3b and c, respectively, over a delay range of −1 to +2 ps. Integrated intensity profiles plotted over the full delay time range are provided in Figure S4. The fitted Gaussian IRFs (light blue), centered at the fitted time zero, are overlaid together with the 95% confidence intervals (error bars) obtained from bootstrap analysis. While the global fit reproduces the integrated photoelectron intensities well, we note that the τ_RI_ and τ_long_ DAS components cannot be cleanly separated, most likely due to significant spectral overlap between both components.

#### 3.1.2 λ*_pump_* = 308 nm

The time-resolved photoelectron spectrum recorded at a pump wavelength of 308 nm in Figure 4a retains the general features observed at 295 nm (Figure 2a): an initially localized signal between 6.0–7.5 eV that decays on an ultrafast time scale into a weaker, more diffuse distribution between 7.5–8.5 eV. However, the time-averaged spectra in Figure 4b reveal subtle differences in the structure of the late-time signal. Near time zero (red trace, −0.15 to +0.15 ps), a broad continuum spanning 6.0–8.5 eV is observed. At intermediate delays (green trace, + 0.6 to + 6 ps), this shifts toward higher binding energies (7.5 8.5 eV), accompanied by a narrowing of the initial distribution. At longer delays (blue trace, +100 to +300 ps), the overall intensity decreases and weak spectral structure emerges between 7.0–8.5 eV. In contrast to the featureless signal observed at 295 nm (Figure 3b), the late-time spectrum exhibits several low-intensity maxima across the 7.0–7.7 eV and 8.1–8.5 eV regions, which may suggest a potential difference in the underlying dynamics and products formed. We note, however, that the overall signal intensity is reduced by approximately 50% relative to 295 nm, reflecting a combination of lower sample pressure during data acquisition and a reduced UV absorption cross-section at 308 nm. To facilitate assignment of the observed features and their associated time scales, a 2D global fit was performed over the 5.5–8.5 eV region of the spectrum.

**Figure 4:**
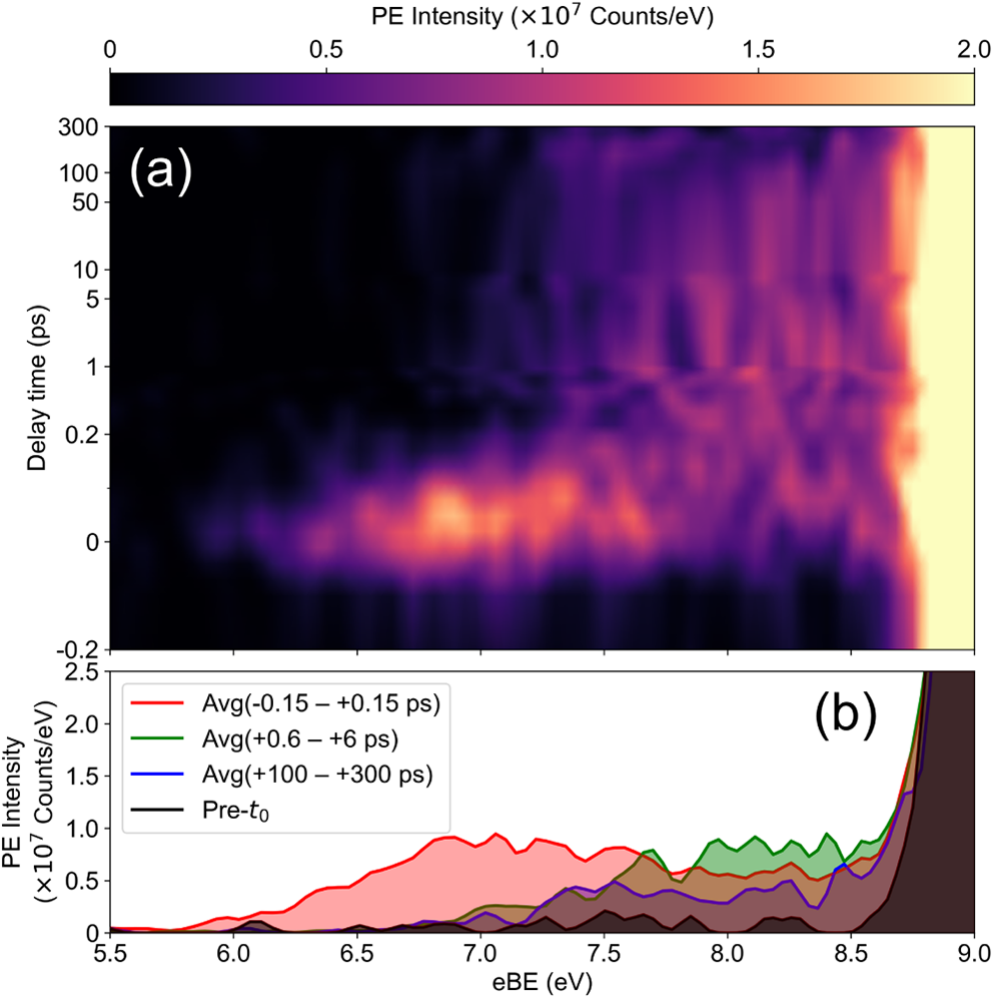
Time-resolved (a) and time-averaged (b) photoelectron spectra of acetaldehyde recorded with a pump wavelength of 308 nm. Note that the delay time axis in (a) is plotted on a mixed linear (up to +0.2 ps)-logarithmic scale. The traces in (b) are averaged (Avg) over specific pump–probe delay time ranges and plotted over the 5.5–9.0 eV binding energy region. All data have a Gaussian filter applied, with a sigma of 1, to highlight the general trend in the flow of signal intensity (population). The acronyms eBE and PE mean electron binding energy and photoelectron, respectively.

In Figure 5, we present the DAS (a) and corresponding fits (b–c) to the integrated intensities from the global fit, plotted over −0.6 to +2.4 ps, capturing the decay of the initial excited state and the rise of the longer-lived signal. Two time components can be reliably extracted. The first ( $\tau_{S_{1}}$ , black in (a)) consists of a positive component, spanning the 5.7–8.0 eV binding energy range, which evolves on a time scale of 40 ± 7 fs. Based on the pump photon energy used in this measurement (4.02 eV) and the calibrated ground-state signal at 10.3 eV (see Figure S1), ionization from the S_1_ state is expected to appear at 6.3 eV. While the low energy onset of this component certainly encompasses this region, the DAS in panel (a) exhibits a maximum intensity near 6.8 eV. The reported UV absorption spectrum of acetaldehyde in Figure 1b shows that the S_1_ $\left(n_{O}\to \pi_{C=O}^{*}\right)$ transition has a spectral width of 0.83 eV centered at 290 nm.^17^ Considering the broad range of excitation energies that can populate the S_1_ state, we assign this component and its associated time constant to the decay of the initially prepared S_1_ state.

**Figure 5:**
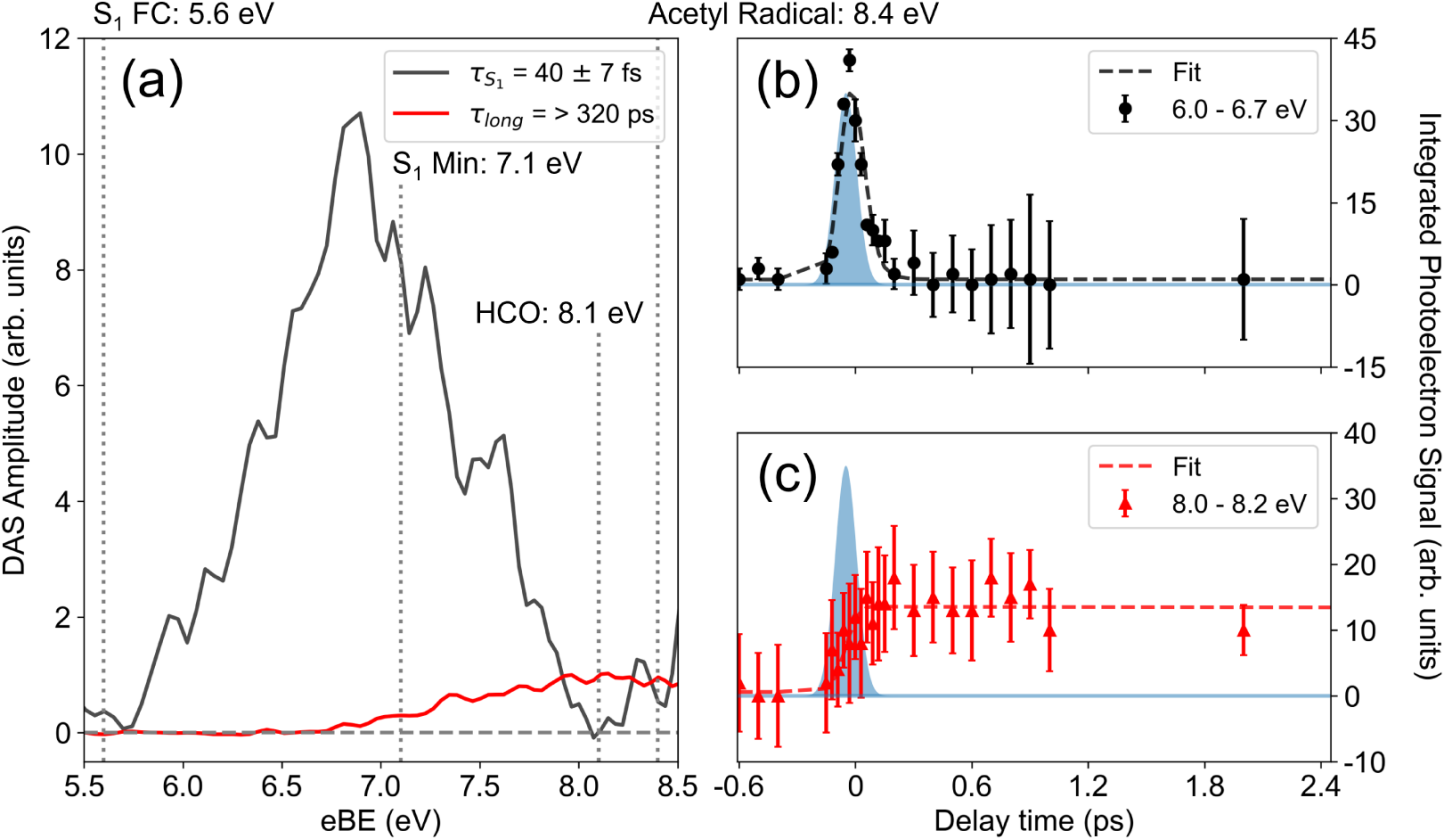
Decay-associated spectra (DAS) and extracted time constants (a), and the corresponding integrated intensity profiles (b–c), for the time-resolved photoelectron spectrum recorded at 308 nm (Figure 2a). Vertical gray dashed lines in (a) indicate the binding energies of the S_1_ Franck–Condon (S_1_ FC) and equilibrium (S_1_ Min) geometries (SA-CASSCF­(14,13), ionization energies calculated at the EOM-IP-CCSD/6-311G** level), as well as expected products: the HCO radical (literature value from ref 44) and the acetyl radical (EOM-IP-CCSD/6-311G**). In panels (b) and (c), integrated intensity profiles are shown over the delay range −0.6 to 2.4 ps, extracted from energy regions of the DAS with minimal overlap between components. Shaded regions represent 95% confidence intervals at each delay time point. All uncertainties were obtained via bootstrap analysis. Further details are provided in the Supporting Information (Section S3).

The second component (τ_long_, red in (a)) is a weaker, positive amplitude feature spanning the 6.7–8.5 eV region and decays on an effectively “infinitely” long time scale (>320 ps) with respect to the full time scale of the measurement. This DAS component exhibits a broad positive distribution that coincides with the low intensity structures observed in the time-resolved photoelectron spectrum (Figure 4b). At this pump wavelength, the formation of the HCO radical is again expected, with a characteristic appearance at 8.1 eV.^44^ However, assignment to HCO alone cannot fully account for all low intensity structures in the time-averaged photoelectron spectrum. While we partially assign this signal to HCO formation, further assignment is deferred to Section 3.2.

The data is well described by two time components, with residuals indicating an adequate reproduction of the signal (see Figure S3). The addition of a third component does not yield any significant improvement in fit quality and is, therefore, not warranted. Overall, the 308 nm results are consistent with those obtained at 295 nm, yielding two comparable time components and well-behaved fits in Figure 5b and c. Nevertheless, differences in signal structure and binding energy distributions at intermediate and long delay times remain evident in the time-averaged photoelectron spectra and require further explanation. Integrated intensity profiles plotted over the full delay time range are provided in the Supporting Information (Figure S4).

### 3.2 Discussion

#### 3.2.1 Dynamics at 295 nm

Based on the photoelectron spectroscopy data, the dynamics initiated by a 295 nm photon can be summarized as follows: excitation promotes population from the ground state to S_1_, which decays on a 60 fs time scale. Decay of the S_1_ state potentially leads to the direct formation of the HCO radical on an ultrafast time scale, and indirectly *via* a longer-lived intermediate species. The intermediate signal is spectrally broad and lacks well-defined structure, making concrete assignment challenging. Based on EOM-IP-CCSD calculations of the ionization energies of the S_1_ Franck–Condon (FC) and equilibrium structures, relaxation within S_1_ may account for the emergence of the ultrafast signal around ∼7 eV (see Figures S5 and 3a), but it cannot explain the full spectral evolution observed toward higher binding energies (>7.1 eV), indicating population transfer out of S_1_ into distinct intermediates and HCO formation.

Considering the direct, ultrafast formation of HCO first, we investigate the excited state pathway by which this is mediated. Figure 6a shows the 2D PES of the S_1_ and S_0_ states along the C–C bond and CCH bending coordinates that characterize the CI seam, calculated at the 2SA-CASSCF­(14,13)/6-311G** level. A minimum energy path (MEP), indicated by the dashed white line, connects the FC region on S_1_ to the CI seam. A contour map of the S_0_ PES is given in Supporting Information (Figure S6). The corresponding state energy difference contour map in Figure 6b reveals a continuous CI seam spanning C–C bond lengths from 2.7 to >5.5 Å and CCH bending angles between 36 and 76°. Along the MEP, the CI is reached at a C–C bond length of 2.7 Å and a CCH angle of 67°, with a barrier of 0.6 eV relative to the FC point. As the experimental pump energy lies ∼0.6 eV above the S_1_ threshold (3.6 eV^17^), this barrier is expected to be readily accessible. Because the MEP is derived from rigid geometry scans, it represents an upper bound to the true barrier, which would likely be reduced by relaxation along the nuclear coordinates. Nevertheless, these calculations confirm that the S_1_/S_0_ CI seam is energetically accessible at 295 nm and likely plays an active role in the observed ultrafast dynamics. We thus suggest that rapid HCO formation proceeds *via* passage over this barrier and along the S_1_/S_0_ CI seam. This is in agreement with the findings and relaxed 2D PES reported by Abma *et al.*, who proposed similar processes following excitation at 262 nm.^26^

**Figure 6:**
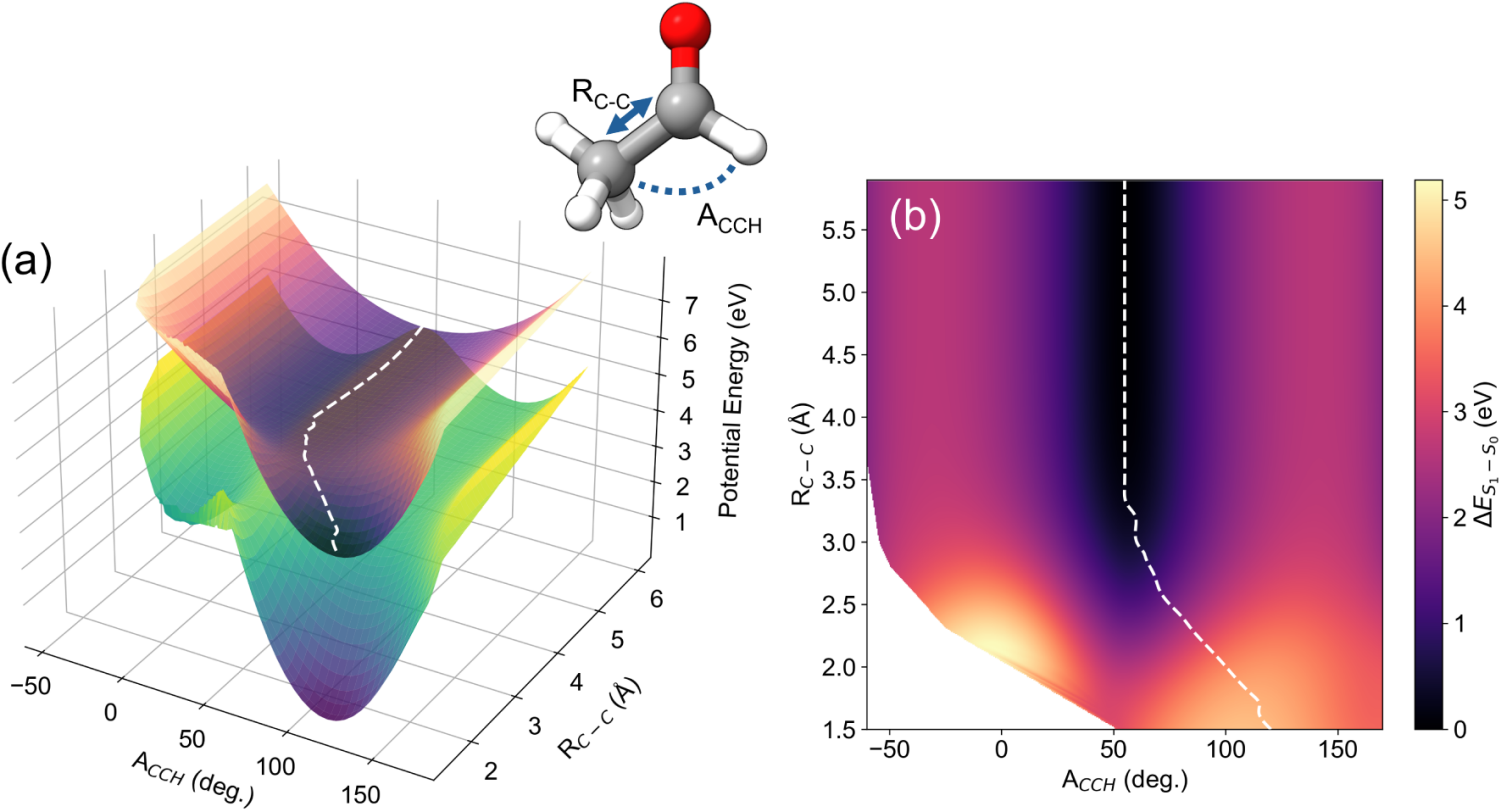
(a) A 2D rigid scan of the potential energy surface of acetaldehyde along the C–C bond elongation (R_C–C_) and CCH angle (A_CCH_) bending coordinates. These coordinates are shown graphically with arrows and dotted lines on the ball-and-stick acetaldehyde model above—gray atoms denote carbon, white atoms are hydrogen, and red is oxygen. The minimum energy path from the Franck–Condon geometry in S_1_ to and through the S_1_/S_0_ CI seam is given by the white dashed line. (b) A 2D contour map showing the difference in potential energy between S_1_ and S_0_ along both sets of nuclear coordinates. Regions of the CI seam are shown in black with the MEP plotted by a white dashed line. All data points were calculated at the 2SA-CASSCF­(14,13)/6-311G** level.

Based on the 2D PES, we expect population transfer through the CI seam onto S_0_, which can account for some longer time scale HCO formation. However, it does not fully explain the rise of the HCO signal on a hundreds-of-picoseconds time scale after the decay of the reaction intermediates. In the DAS shown in Figure 3a, a spectral component with a time constant of 4.0 ps, associated with some reaction intermediates, is observed to follow the decay of the S_1_ state, although its precise identity remains unclear. The time-resolved photoelectron spectrum in Figure 2a and the negative contribution of the τ_RI_ DAS component in Figure 3b show that this intermediate signal shifts over time toward ∼8 eV binding energy, suggesting that its decay feeds population into the formation of HCO at a later time. We therefore examine the nature of these reaction intermediates.

One possible assignment for these reaction intermediates involves structures formed on the T_1_ state following ISC from S_1_. SOC matrix elements, calculated at the 3SA-CASSCF­(14,13)/6-311G** level, range between 10–20 cm^–1^ at S_1_/T_1_ crossing points along the C–C 1D PES cut (see Figure S7). Given these small magnitudes, efficient ISC on the time scale of the S_1_ lifetime (60 fs) is unlikely. Indeed, Landau–Zener analysis (see Section 5 of Supporting Information for methodology) suggests that ISC could occur on time scales of 2–16 ps—much longer than our observed rate. At 262 nm, Abma *et al.* assigned a broad, structureless signal spanning 5.3–7.6 eV to ionization of CH_3_ roaming intermediates, supported by calculated ionization energies of possible roaming structures sampled on their S_0_ PES.^26^ EOM-IP-CCSD calculations performed from sampled geometries on the S_0_ PES in Figure 6a (see Figure S6) predict a similarly broad range of ionization energies (6.0–8.3 eV), consistent with the signal observed here and attributed to reaction intermediates. Although both the experimental feature and calculated energies are shifted to slightly higher binding energies compared to the 262 nm measurements, the spectral characteristics and temporal behavior remain consistent. On this basis, the observed signal is expected to be largely dominated by CH_3_ roaming intermediates, but likely contains contributions from conventional TS dissociation. We therefore treat the extracted 4.0 ps time constant as a characteristic time scale associated with population evolution following passage through the S_1_/S_0_ CI seam, rather than as a pure CH_3_ roaming lifetime. Formation of CH_3_ roaming intermediates is consistent with the topology of the S_0_ potential energy surface near the S_1_/S_0_ CI seam (Figure 6a), which is relatively flat and conducive to roaming following internal conversion (IC). These findings suggest that HCO formation at 295 nm proceeds via both the conventional TS mechanism on S_1_ and CH_3_ roaming dynamics on S_0_. We thus infer concomitant formation of a CH_3_ cofragment, with molecular products appearing at longer times, consistent with prior energy-resolved measurements.^19–23,27,28^

While the assignments are consistent, the 4.0 ps lifetime at 295 nm is significantly shorter than the roaming lifetime extracted at 262 nm (190 ps^26^), likely reflecting differences in the initially populated vibrational modes despite broadly similar overall dynamics. At 262 nm, and assuming a 5 nm pump bandwidth, excitation is expected to populate CH_3_ deformation and aldehyde C–H bending modes as reported in the high-resolution UV absorption spectroscopy work of Limão-Vieira and co-workers^17^ and shown in Figure 1b. These modes drive nuclear motion that extends the C–C bond and reduces the CCH bending angle, thereby facilitating access to the CI seam depicted in Figure 6. The nuclear displacement vectors of the modes accessed are shown in Figure S8. In contrast, excitation at 295 nm, and assuming the same pump bandwidth, preferentially populates modes, also shown in Figures 1b and S8, localized on the aldehyde moiety, including C–H bending and stretching coupled with C=O stretching. Although these motions also promote CI access, the additional C=O stretching contribution at 295 nm may alter subsequent dynamics on S_0_. Our current interpretation is that this mode promotes more rapid evolution away from the roaming region of the potential energy surface, favoring radical and molecular product formation and thus reducing the apparent roaming lifetime. Given that differences in vibrational excitation represent the primary experimental distinction between the two datasets, such effects may contribute to the shorter lifetime observed at 295 nm. Further investigation of this possibility would require systematic wavelength-dependent measurements across the absorption band and nonadiabatic dynamics calculations that explicitly assess the role of vibrational excitation.

#### 3.2.2 Dynamics at 308 nm

The dynamics observed at 308 nm are less straightforward than at 295 nm, but remain closely related. The time-resolved photoelectron spectrum in Figure 4a exhibits a similarly short-lived S_1_ population (40 fs) and broadly comparable long-lived spectral features, indicating that the same primary relaxation pathways (i.e., the formation of HCO) are active. However, subtle differences suggest the presence of additional contributions. The most prominent distinction is the emergence of low intensity spectral structure in the intermediate and late-time signals between 7.0–8.5 eV in Figure 4b, compared to the featureless late-time signal at 295 nm in Figure 2b. This long-lived signal appears to decay over a tens-of-picoseconds time scale as shown in Figure 4a. As established at 295 nm, relaxation within S_1_ alone cannot account for the observed shift of spectral weight toward higher binding energies (>7.1 eV), indicating that the dynamics involve population transfer into additional intermediate or product channels.

Previous studies at this wavelength, as described in Figure 1a, have proposed competing pathways involving triplet state dynamics, CH_3_ roaming, and H-atom roaming.^19,22,27,28^ As discussed above, the calculated SOC matrix elements between S_1_ and T_1_ along the C–C bond coordinate are small and do not support efficient ultrafast ISC, allowing triplet pathways to be discounted as a major contributor. In contrast, multiple lines of evidence support CH_3_ roaming as an active photodissociation pathway. Reported branching ratios at 308 nm indicate significant CH_3_ roaming contributions,^22,27,28^ and the flat topology of the S_0_ surface near the S_1_/S_0_ CI seam, shown in Figure 6a, remains conducive to roaming following IC. Most compellingly, the time-resolved photoelectron spectrum at 308 nm in Figure 4a retains the same broad spectral characteristics observed at 295 nm, where CH_3_ roaming has been established as the dominant pathway. Prior EOM-IP-CCSD calculations of prospective S_0_ CH_3_ roaming intermediates (see Figure S6) collectively reproduce the principal binding energy range of the longer-lived spectral features observed here. Taken together with the overall extracted dynamical time scales, the assignment of HCO formation in the DAS (Figure 5a), and parity with XUV-TRPES work performed at shorter UV pump wavelengths (262 nm^26^ and 295 nm; this work), these findings suggest that CH_3_ roaming continues to play a major, and potentially dominant, role in the photodissociation dynamics at 308 nm.

However, this mechanism alone does not fully account for the structured signal emerging between 7.0–8.5 eV. Although H-atom roaming is expected to be a minor channel, it remains comparatively unexplored in acetaldehyde. We therefore consider its potential spectral signatures. The products of H-atom roaming include acetyl radicals (CH_3_CO) and H atoms, as well as CO + CH_4_ pairs. The ionization energies of the molecular products overlap with the ground state acetaldehyde signal (see Figure S1), complicating their signal isolation. The ionization energy of H is a similar case with a measured ionization energy of 13.6 eV.^49^ The ionization energy of the acetyl radical has been reported from electron impact measurements as 8.0 ± 0.17 eV;^50^ however, to our knowledge, no photoionization measurements are available. Our EOM-IP-CCSD/6-311G** calculations on an MP2-optimized acetyl radical structure, shown in Figure 7, predict an ionization energy of 8.4 eV, in good agreement with the electron impact value and coincident with the late-time spectral structure observed in Figure 4b.

**Figure 7:**
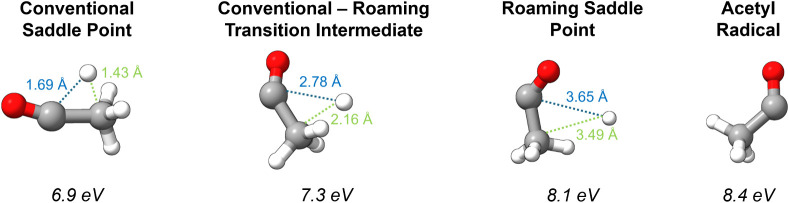
Ionization energies of H atom roaming intermediates calculated at the EOM-IP-CCSD/6-311G** level. Coordinates for each geometry was taken from ref 42. Blue dashed lines denote the bond length between initially bonded fragments. Green dashed lines denote the bond length from the H atom involved in the formation of the molecular products (CO + CH_4_) and the CH_3_ fragment. For each ball-and-stick structure, white atoms are hydrogen, red is oxygen, and gray is carbon.

If H-atom roaming occurs, the spectrum should also contain contributions from roaming intermediates, whose structures and ionization energies remain largely unknown. To constrain these contributions, we drew on analogous structures in formaldehyde. Shepler *et al.* identified ground-state saddle points corresponding to conventional H-atom dissociation, H-atom roaming, and a transition structure connecting the two pathways.^42^ These geometries, shown in Figure 7, were adapted by substituting the secondary hydrogen atom with a CH_3_ group to approximate acetaldehyde-like structures, and their ionization energies were computed at the EOM-IP-CCSD level. The resulting values, 6.9, 8.1, and 7.3 eV, respectively, show that the transition intermediate and roaming saddle point overlap strongly with photoelectron intensity between intermediate and late delay times, while the conventional dissociation saddle point lies outside of this range. Although these estimates are based on rigid, non-optimized structures and should be interpreted qualitatively, their agreement with the observed energy window, together with the acetyl radical signal, suggests that H-atom roaming may contribute to the dynamics at 308 nm.

The excited state pathways leading to these intermediates remain unclear. Rigid 1D cuts of the PES along the C–H coordinate (see Figure S9) indicate a loosely bound region at extended C–H bond lengths on the S_1_ surface that could, in principle, support H-atom roaming, but this region appears energetically inaccessible at 308 nm. By analogy, CASSCF-level 2D PES of acetone, an analogue of acetaldehyde with an additional CH_3_ group, has revealed an additional S_1_/S_0_ CI that facilitates access to roaming pathways.^51^ Whether similar features exist in acetaldehyde remains an open question. Given the limited theoretical characterization of H-atom roaming in this system, a comprehensive investigation lies beyond the scope of the present work. Nevertheless, the overlap between calculated ionization energies and the observed spectral features provides tentative evidence that H-atom roaming pathways may be accessed at 308 nm, although a definitive assignment cannot yet be made.

Finally, the driving force behind the 308 nm dynamics may again be rooted in mode selectivity. Within the 5 nm pump bandwidth, the dominant excited vibrational motions (Figures 1b and S8) involve aldehyde C–H stretching and bending, which naturally promote C–H elongation and dissociation.^17^ Although this might appear to disfavor CH_3_ roaming, both previous studies at 308 nm^19,22,27,28^ and the similarity of the time-resolved photoelectron spectrum in Figure 4a to the 295 and 262 nm results indicate that it remains an active pathway. We propose that the differences in dynamics between pump wavelengths may arise from a secondary H-atom roaming pathway, enhanced by the preferential population of C–H stretching modes, which may steer a fraction of the excited state population into a distinct region of the S_1_ surface associated with H-atom roaming rather than CH_3_ roaming. While a conventional TS mechanism along the S_1_/S_0_ CI seam cannot be definitively resolved from the present data, the absence of a clear negative contribution in the $\tau_{S_{1}}$ DAS component in Figure 5a suggests that its contribution is minor or obscured by reduced signal. We therefore treat this as a minor contributor to the overall dynamics. Although branching ratios cannot be extracted from the present measurements due to our inability to resolve individual reaction pathways uniquely, the present observations remain qualitatively consistent with the mechanistic picture proposed by Lee *et al.*, suggesting that CH_3_ roaming remains the dominant dissociation mechanism at 308 nm.^28^

#### 3.2.3 Astrochemical Implications

Our results, together with those of Abma *et al.*,^26^ demonstrate that CH_3_ roaming appears to be an active and potentially dominant photodissociation mechanism for acetaldehyde across the center of the $n_{O}\to \pi_{C=O}^{*}$ absorption band. Because roaming bypasses high energy barriers, it provides an efficient route to radical formation under UV-irradiated and thermally cold conditions, such as protoplanetary disk midplanes and dense molecular clouds including Sgr B2. The growing number of organic species for which roaming-mediated photochemistry has been observed—acetone,^52^ propionaldehyde,^53^ methyl formate,^54^ and methylamine,^55^ among others—further supports the view that roaming is a widespread dynamical motif in interstellar photochemistry.

Importantly, we suggest that the roaming products in acetaldehyde are wavelength- and mode-dependent. At 262 and 295 nm, roaming yields HCO and CH_3_ radicals, while at 308 nm we find tentative evidence for the formation of the acetyl radical (CH_3_CO) and a H atom. These radicals have distinct chemical fates in the ISM: CH_3_ drives carbon-chain growth^56,57^ and forms a key interstellar gas thermometer—methyl cyanide (CH_3_CN),^58^ while HCO participates in the foundations of prebiotic chemistry such as the barrierless formation of formamide (NH_2_CHO).^59,60^ Although the acetyl radical has yet to be detected in the ISM at the time of writing, it is a potential intermediate for building peptide-like molecules based on similar reactions with HCO. Reactions between CH_3_CO and NH_2_ could plausibly lead to acetamide—already observed in Sgr B2 ^61^—in the gas phase or on the surface of icy dust grains. Taken together, this suggests that roaming in acetaldehyde can feed molecular growth pathways leading toward increasingly complex organic molecules in regions where acetaldehyde is known to be abundant.

The tentative detection of the acetyl radical here also suggests the formation of a photolysis product of acetaldehyde that is not typically included in current astrochemical models and merits further investigation. If confirmed to be present, such a pathway would warrant consideration in future astrochemical reaction networks, particularly in UV-rich interstellar environments such as Sgr B2, where roaming-mediated dissociation pathways may be active.

## 4 Conclusions

In this work, we have investigated the wavelength-dependent photodissociation dynamics of acetaldehyde using time-resolved photoelectron spectroscopy with a universal extreme ultraviolet probe supported by *ab initio* electronic structure calculations. Excitation at 295 nm is suggested to produce roaming dynamics that closely resemble those observed at 262 nm by Abma *et al.*,^26^ yielding HCO radical products via both conventional transition-state-mediated dissociation through the S_1_/S_0_ conical intersection and CH_3_ roaming processes on S_0_. The lifetime associated with the roaming intermediates is shorter than that reported by Abma *et al.* (4.0 ps here, 190 ps at 262 nm), which we attribute to additional excitation of the C=O stretching mode on S_1_ at 295 nm that may perturb the roaming complex away from the flat region of the S_0_ potential energy surface.

In contrast, excitation at 308 nm leads to somewhat different dynamics. The emergence of a structured signal between 7.0–8.5 eV at intermediate and late delay times, supported by EOM-IP-CCSD calculations, suggests that additional pathways beyond CH_3_ roaming may become accessible at this wavelength, with H-atom roaming representing a plausible contribution through the formation of the acetyl radical. The vibrational modes accessed at 308 nm preferentially drive C–H bond extension, which may provide a route toward such dynamics. Taken together, these observations suggest that roaming in acetaldehyde may exhibit a degree of mode selectivity, with the vibrational energy deposited in S_1_ influencing access to different regions of the S_1_ surface and giving rise to wavelength-dependent photodissociation behavior. However, consistent with previous studies, CH_3_ roaming appears to be the dominant dissociation pathway at both 295 and 308 nm.

Future studies should aim to directly detect and quantify all roaming-derived radical and molecular products, for example, via time-resolved mass spectrometry or photoelectron–photoion coincidence measurements, in order to establish wavelength-dependent branching ratios and better inform astrochemical modeling of acetaldehyde. In addition, a more detailed computational investigation of the H-atom roaming pathway is required to elucidate the excited state channels through which it may be accessed. Such work would strengthen the mechanistic understanding of how mode-selective excitation governs access to distinct roaming pathways and provide definitive photoelectron spectral signatures for comparison with the 308 nm data presented here.

## Supplementary Material



## Notes

Time-of-flight to electron binding energy calibration, global fitting procedure details, simulated spectra, and residuals, longer delay time intensity profiles, procedures for bootstrapping methods, vibrational modes of the optimized S_0_ equilibrium geometry, EOM-IP-CCSD calculations of S_0_ and S_1_ equilibrium structures and of representative roaming intermediates, Franck–Condon analysis calculations and 1D C–C potential energy surface, active vibrational modes at 262, 295, and 308 nm, and 1D C–H potential energy surface (PDF)
