# Supplementary material for: Wherever I May Roam: A Time-Resolved Wavelength-Dependent Study of the Roaming Dynamics in Acetaldehyde
Source: ACS Earth Space Chem. 2026 Jun 23;10(7):1805–16. doi: 10.1021/acsearthspacechem.6c00134 (PMC13383833; doi:10.1021/acsearthspacechem.6c00134)
Supplement: Supplementary file 1 [file sp6c00134_si_001.pdf]

Wherever I May Roam: A Time-Resolved Wavelength-Dependent Study of the Roaming Dynamics in Acetaldehyde
Supporting Information

Derri J. Hughes[1,*], Michael A. Parkes[2], Richard T. Chapman[3], M. Nrisimhamurty[3], Emma Springate[3], James O. F. Thompson[3], Tiffany Walmsley[3], Yu Zhang[3], and Russell S. Minns[1,*]

[1]School of Chemistry and Chemical Engineering, University of Southampton, University Road, Highfield, Southampton, SO17 1BJ
[2]Department of Chemistry, University College London, 20 Gordon Street, London, WC1H 0AJ, United Kingdom.
[3]Central Laser Facility, STFC Rutherford Appleton Laboratory, Didcot, Oxfordshire OX11 0QX, United Kingdom.
[*]Corresponding authors: Derri J. Hughes (`d.j.hughes@soton.ac.uk`) and Russell S. Minns (`r.s.minns@soton.ac.uk`)

1 Photoelectron Calibration

The binding energy of the detected photoelectrons was calibrated by comparison with the He(I) photoelectron spectrum of acetaldehyde and N_2. [1, 2] Table S1 lists our calibrated signals of ground state acetaldehyde. For both pump wavelengths (295 and 308 nm), we performed separate calibrations due to the difference in appearance of the observed signals in the time domain between measurements. We have plotted our calibrated photoelectron spectra in Figure S1 below. Although both experiments were performed with a acceptance cone voltage of approximately 95 V, minor fluctuations in this parameter have contributed to variations in the observed width of the photoelectron ground state signals, subsequently shifting the peaks of some signals between measurements. However, these shifts are well-within the error in the fitted probe energy ($\pm$ 0.67 eV) and do not affect our observations or fits of the dynamics.

Table S1: Comparison between the reported ground state acetaldehyde ionisation bands and those obtained in this work. Reference ionisation bands have been taken from ref [1] (acetaldehyde) and ref [2] (N_2).

Band	Reported Energy (eV)	This work (295 nm, eV)	This work (308 nm, eV)
1st	10.2	10.3	10.3
2nd	13.2	13.2	13.0
3rd	14.1	14.7	14.7
4th	15.3	15.3	15.3
5th	16.4	16.4	16.4
N_2 (B State)	18.75	18.75	18.75

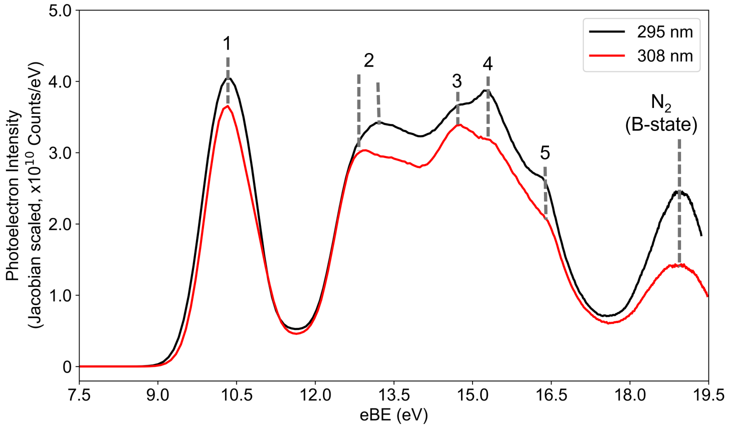

Figure S1: The calibrated pre-time-zero, ground state spectrum of acetaldehyde collected at pump wavelengths of 295 nm (black trace) and 308 nm (red trace) respectively. The numbering refers to the band labelling in Table S1.

2 Global Fitting Procedure

In order to extract dynamic information from the time-resolved photoelectron spectra (both spectral components and time constants), we performed a 2D least-squares fit to generate a decay-associated spectrum (DAS). [3] Each spectrum was modelled using a single Gaussian instrument response function (IRF):

$$g(t) = A_{\mathrm{IRF}} \exp\left(-(\frac{t-t_0}{2\sigma_{\mathrm{IRF}}})^2)\right) \tag{1}$$

where A_{IRF} is the amplitude of the Gaussian, t is the pump-probe delay time, t_0 is the time-zero (maximum pump-probe overlap), and σ_{IRF} is the temporal resolution (pulse duration). This IRF was convolved with a sum of exponential decay functions to produce the full model:

$$S(E,t) = \sum_i A_i(eBE)\, e^{-\lambda_i(t-t_0)} \otimes g(t) \tag{2}$$

where λ_i is the ith decay constant and the time constants are $\tau_i = 1/\lambda_i$. The DAS thus represents a set of time constants associated with discrete binding energy regions of the photoelectron spectrum.

In Figs. S2 and S3, we plot the reconstructed time-resolved photoelectron spectrum and the residuals of the fit for both wavelength measurements. To determine the quality of the fit, we compared the reconstructed spectrum to the residuals. If there appears to be no coherent time-resolved patterns, then the number of exponential decay terms used to reconstruct the spectrum is deemed appropriate. In the case of our data, 3 terms were required to adequately reconstruct the 295 nm data, and 2 terms for the 308 nm data.

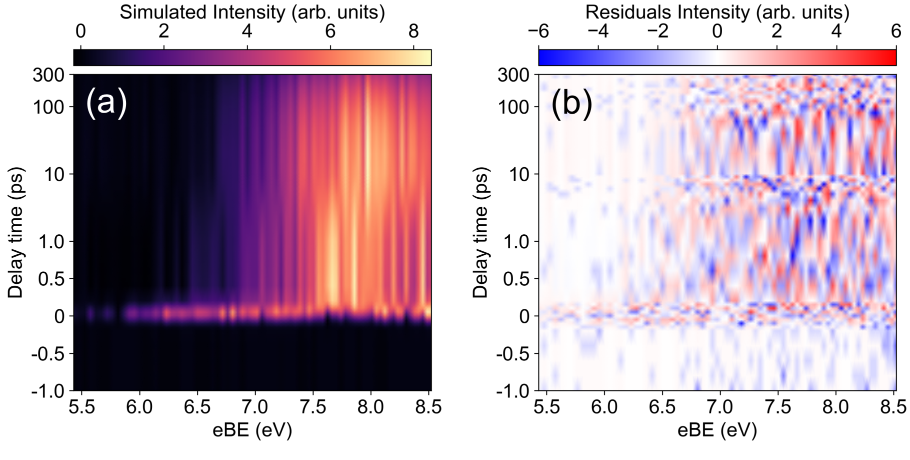

Figure S2: (a) Simulated time-resolved photoelectron spectrum at 295 nm from the global fit. (b) The resultant residuals. Both delay time axes are presented on a mixed linear/logarithmic scale to analyse the spectrum and residuals out to the full temporal extent of the data.

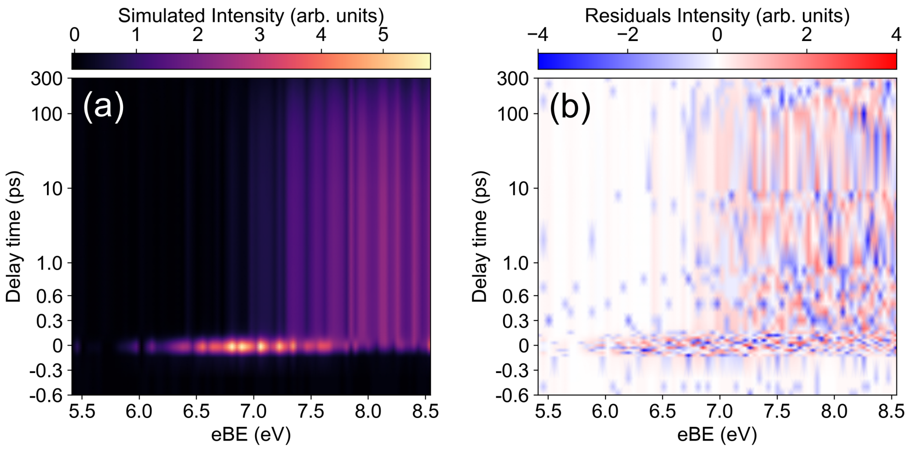

Figure S3: (a) Simulated time-resolved photoelectron spectrum at 308 nm from the global fit. (b) The resultant residuals. Both delay time axes are presented on a mixed linear/logarithmic scale to analyse the spectrum and residuals out to the full temporal extent of the data.

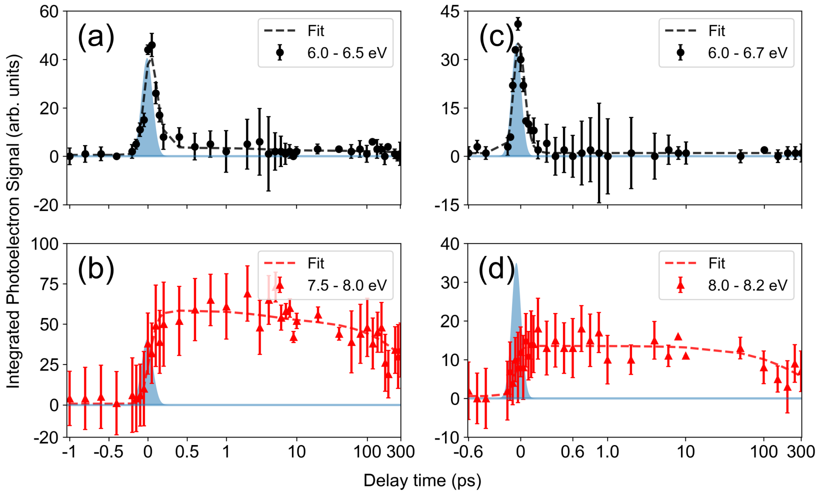

Figure S4: Integrated intensities from the global fitting procedure plotted over the full pump-probe delay time range. (a – b) are the fits extracted from the 295 nm dataset and (c – d) are from the 308 nm dataset. Error bars at each delay time represent the 95% confidence intervals of the integrated intensity as obtained from Bootstrap analysis. Blue Gaussian defines the Gaussian IRF for each wavelength, with widths of 70 fs for 295 nm and 60 fs for 308 nm. Note, the delay time axes are plotted on a mixed linear (up to 1 ps)–logarithmic scale.

3 Bootstrapping Method

3.1 Errors on Global Fit Values

To obtain confidence regions on our extracted global fit parameters, we used a parametric Bootstrapping approach. In this, 1000 datasets were constructed by adding a random, Gaussian-distributed amount of noise to each data point in the spectrum. In this, the width of said Gaussian was made equal to the ratio of the sum of the residuals to the sum of the data. The global fit was performed on each of the 1000 datasets independently of one another. The fitted parameters for each energy bin were investigated and the 95% confidence interval was obtained by fitting a Gaussian to a histogram of the results. The original global fit was then performed once again to which the initial fitted parameters were constrained by an upper and lower bound supplied by the bootstrapped error. This allowed us to find the true fitted parameter. The error thus reported was calculated by taking the square root of the diagonal of the outputted covariance matrix from the fitting algorithm.

3.2 Errors on Integrated Delay Points

The data used in fitting is a sum of averaged data, averaged every 20 delay cycles. Our experimental data consisted of 2300 cycles for 295 nm and 980 cycles for 308 nm, and therefore contains 115 and 49 compounded spectra respectively. In order to obtain 2σ uncertainties on each delay point in our reported intensity profiles, we took a case resampling bootstrapping approach [4]. Simply, we took each 20-cycle averaged spectrum, performed a *time-of-flight* to *binding energy* calibration, and integrated over the binding energy range of interest. The 95% confidence intervals were then obtained by resampling the integrated data values 1000 times and taking twice the standard deviation of the bootstrapped values. The only consideration in this method is that each averaged spectrum must be subtracted from the one recorded immediately before it, to obtain a spectrum representing only the most recent 20 cycles and not the cumulative signal.

4 Supporting Calculations

4.1 Vibrational Normal Modes of S_0 Equilibrium Structures

Table S2: Vibrational normal modes of S_0 optimised acetaldehyde, their frequencies, and symmetries. Calculated at the CASSCF(14,13)/6-311G** level.

Mode	Frequency (cm^{-1})	Symmetry
1	155.36	A''
2	525.91	A'
3	820.37	A''
4	931.46	A'
5	1179.14	A'
6	1188.22	A''
7	1429.48	A'
8	1485.37	A'
9	1496.80	A''
10	1511.40	A'
11	1808.73	A'
12	2934.89	A'
13	2995.83	A''
14	3041.38	A'
15	3061.97	A'

4.2 EOM-IP-CCSD Calculations

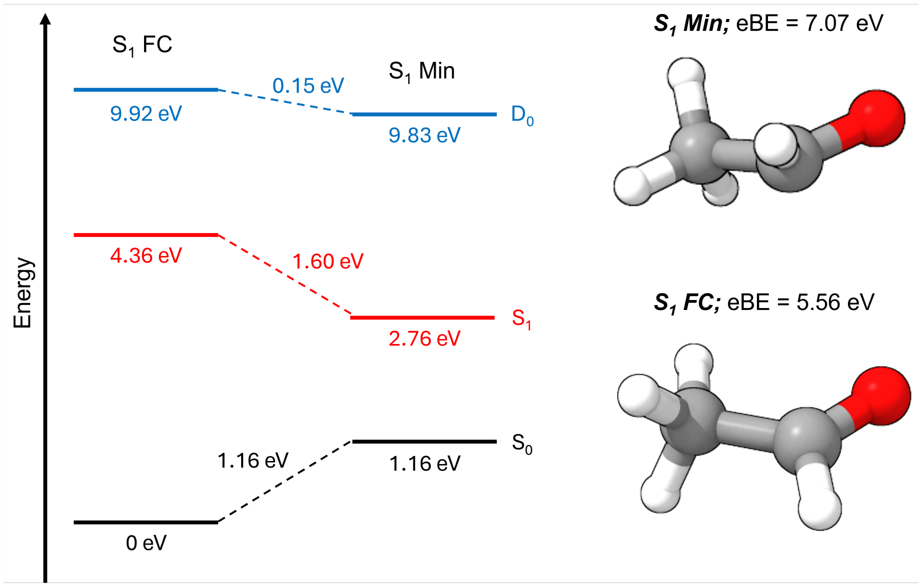

Figure S5: Results of EOM-CCSD and EOM-IP-CCSD calculations of SA-CASSCF(14,13)/6-311G** Franck-Condon (S_1 FC) and equilibrium (S_1 Min) structures. Energies of the S_0 and S_1 states were calculated at the EOM-CCSD level and energies of D_0 at the EOM-IP-CCSD level. All calculations were performed using the 6-311G** basis set. Electron binding energies (eBEs) were formally calculated *via* eBE $= E_{D_0} - E_{S_1}$. For each ball and stick structure, white atoms are hydrogen, red is oxygen, and grey is carbon.

Table S3: Cartesian coordinates of the S_1 Franck–Condon (FC) and equilibrium (S_1 Min) structures. Coordinates are given in Å.

	FC				S_1 Min		
Atom	x	y	z	Atom	x	y	z
O	-1.223959	0.366606	0.000000	O	-1.155154	0.411865	0.544277
C	0.938853	-0.696375	0.000000	C	0.955211	-0.703507	-0.045342
C	-0.014039	0.468035	0.000000	C	-0.026499	0.461766	-0.211500
H	1.597243	-0.649689	0.891176	H	1.294300	-0.778415	1.015088
H	0.385637	-1.651623	0.000000	H	0.459618	-1.663637	-0.295343
H	1.597243	-0.649689	-0.891176	H	1.814783	-0.566771	-0.687072
H	0.460675	1.459567	0.000000	H	0.399396	1.485528	-0.320108

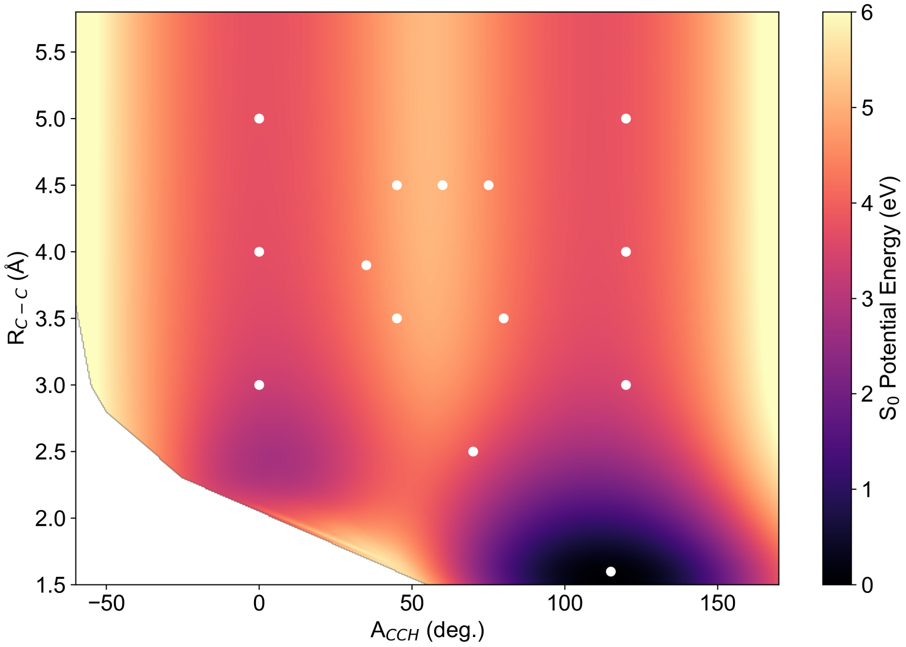

Figure S6: S_0 potential energy surface as a function of the C-C bond length ($R_{\text{C-C}}$) and CCH bending angle (A_{CCH}), calculated at the SA-CASSCF(14,13)/6-311G** level. White markers indicate the sampled geometries used for EOM-IP-CCSD calculations of prospective CH_3 roaming intermediates.

4.3 Intersystem Crossing Timescales

To estimate a lower bound for the timescales of intersystem crossing (ISC) at 295 nm, we first calculate the gradients (F) of each state of interest from the 1D potential energy surface around the minimum energy crossing point (MECP). Here, we only consider the lower S_1/T_1 MECP at a C-C bond extension of 2 Å due to it being the most energetically favourable crossing at both 295 nm. We show this graphically in Figure S7. From here, the difference gradient (ΔF) can be calculated *via* $|F_{S_1} - F_{T_1}|$.

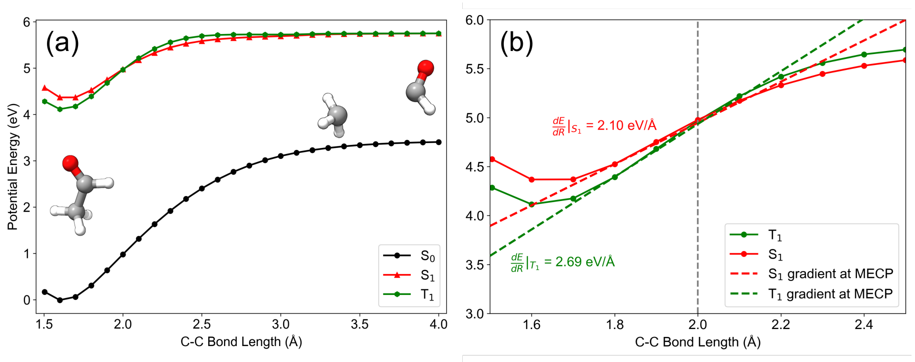

Figure S7: (a) 1D potential energy surface along the C-C bond. For each ball and stick structure, white atoms are hydrogen, red is oxygen, and grey is carbon. (b) A cut of (a) focusing on the S_1/T_1 minimum energy crossing point (MECP) at 2Å indicated by the grey dashed line. The respective gradients of each state around the MECP are given in red (S_1) and green (T_1) dashed lines. Calculated at the 3SA-CASSCF(14,13)/6-311G** level of theory.

As we are considering a crossing between states, a Landau-Zener approximation provides the necessary framework to calculate the probability of crossing per pass. By pass, we mean the traversal of the vibrational wavepacket within the region of the MECP. The Landau-Zener probability per pass (P_{pass}) is given by

$$P_{pass} = 1 - \exp\left(-\frac{2\pi V^2}{\hbar v \Delta F}\right) \tag{3}$$

where V is the diabatic coupling energy between S_1 and T_1 and v is the nuclear velocity along the crossing coordinate. Here, we use the spin-orbit coupling matrix element at the MECP as our given value of V (20 cm^{-1} = 2.48×10^{-3} eV). As the MECP exists in a region of the potential energy surface that can reasonably be considered harmonic, motion along the surface can thus be treated as oscillatory. We therefore estimated nuclear velocities from a harmonic oscillator model of the C-C stretching coordinate. The calculated vibrational frequency of this mode, at the CASSCF(14,13)/6-311G** level is 931 cm^{-1} (see Table S2).

Characteristic maximum nuclear velocities (v_{max}) were thus obtained by

$$v_{max} = \omega A \tag{4}$$

where ω is the angular frequency of the mode ($=2\pi c\tilde{\nu}$ where c is the speed of light in cm s^{-1} and $\tilde{\nu}$ is the vibrational frequency in cm^{-1}), and A is the local oscillation amplitude in Å. A range of values from 0.05 – 0.5 Å were considered, spanning from modest to strongly vibrationally excited regimes, and yielded no considerable dependence on the value of P_{pass} for any given value of A.

Using the calculated values for P_{pass}, an effective timescale for ISC ($\tau_{ISC,\text{eff}}$) between S_1 and T_1 could be estimated as

$$\tau_{ISC,\text{eff}} \sim \frac{T_{osc}/2}{P_{pass}} \tag{5}$$

where T_{osc} is the oscillation period of the aldehyde C-C stretching mode (11.4 fs) and under the assumption that the MECP is reached twice per C-C bond oscillation.

Table S4 details the output of these calculations for varying values of A. Even for the slowest nuclear velocities in which crossing between states would be more likely, P_{pass} does not surpass 0.0113 (or 0.037 within 3 passes of the MECP, corresponding to the measured lifetime of the S_1 state), resulting in a lower bound for $\tau_{ISC,\text{eff}}$ of 1.6 ps. Across all sampled nuclear velocities, the following relationship remains consistent: $\tau_{ISC,\text{eff}} >> \tau_{S_1}$. This pronounced mismatch of timescales, albeit within this crude approximation, indicates that ultrafast ISC cannot compete with the non-radiative decay of the S_1 state on the observed timescale of its decay (60 fs), and it unlikely to be a major contributor to the observed dynamics at 295 nm.

Table S4: P_{pass} and $\tau_{ISC,\text{eff}}$ values for varying nuclear velocities (v_{max}).

A (Å)	v_{max} (Å/fs)	P_{pass}	P_{pass} within τ_{S_1}	$\tau_{ISC,\text{eff}}$ (ps)
0.05	0.009	0.0113	0.037	1.6
0.1	0.018	0.0057	0.019	3.2
0.2	0.035	0.0028	0.010	6.3
0.3	0.053	0.0019	0.006	9.5
0.4	0.072	0.0014	0.005	12.6
0.5	0.088	0.0011	0.004	15.8

4.4 Active Vibrational Modes

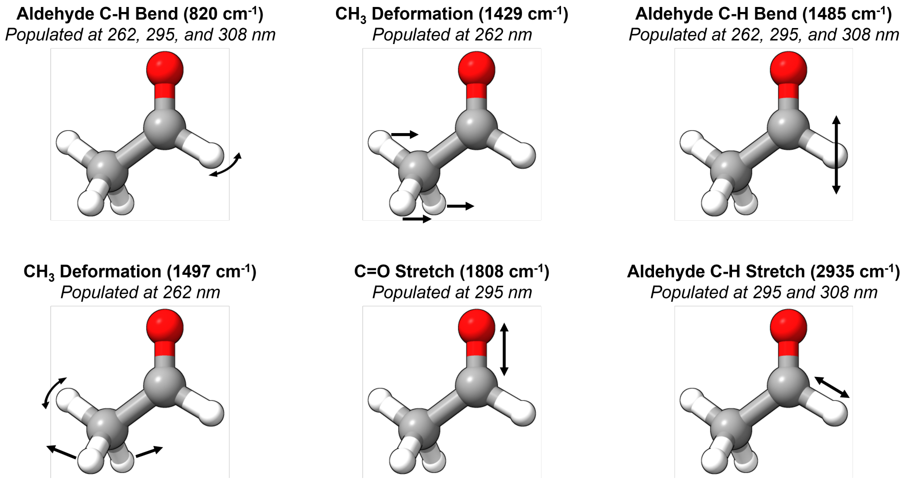

Figure S8: Vibrational modes populated upon excitation by 262, 295, and 308 nm respectively as reported by Limão-Vieira and co-workers. [5] Mode frequencies relate to those calculated in Table S2. Black arrows refer to the dominant displacement vector associated with each mode. For each ball and stick structure, white atoms are hydrogen, red is oxygen, and grey is carbon.

4.5 1D C-H Potential Energy Surface

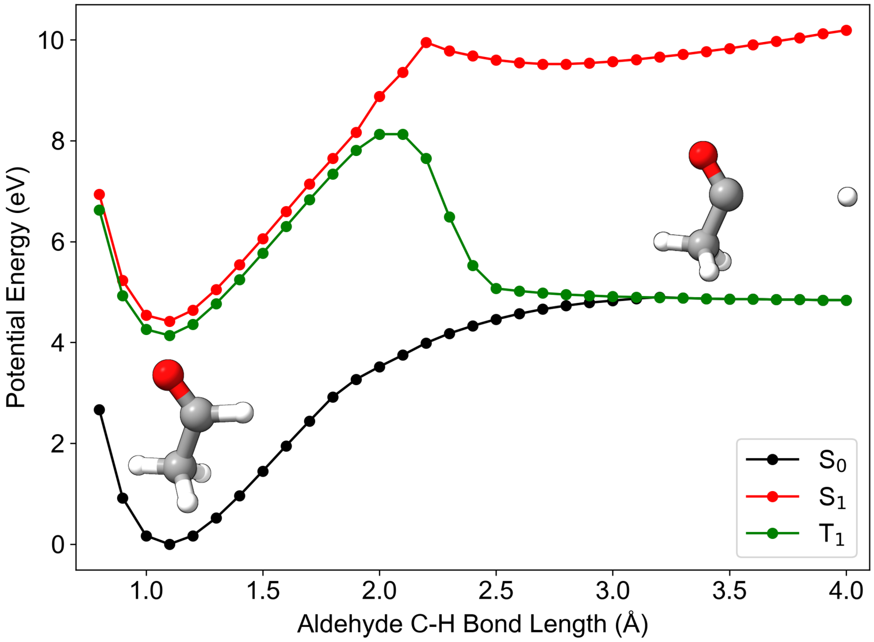

Figure S9: 1D potential energy cuts as a function of aldehyde C-H bond length on the S_0, S_1, and T_1 states. The 1D cuts have been calculated using the S_0 optimised geometry at the 3SA-CASSCF(14,13)/6-311G** level. Ball and stick models show the start and final geometries – grey atoms are carbon, white atoms are hydrogen, and red atoms are oxygen.

References

(1) W.-C. Tam, D. Yee and C. Brion, *Journal of Electron Spectroscopy and Related Phenomena*, 1974, **4**, 77–80.

(2) D. W. Turner, C. Baker, A. D. Baker and C. R. Brundle, *Molecular Photoelectron Spectroscopy*, John Wiley & Sons, Chichester, England, 1970.

(3) J. S. Beckwith, C. A. Rumble and E. Vauthey, *International Reviews in Physical Chemistry*, 2020, **39**, 135–216.

(4) A. C. Davison and D. V. Hinkley, *Cambridge series in statistical and probabilistic mathematics: Bootstrap methods and their application series number 1*, Cambridge University Press, Cambridge, England, 1997.

(5) P. Limão-Vieira, S. Eden, N. Mason and S. Hoffmann, *Chemical Physics Letters*, 2003, **376**, 737–747.
